# Collaboration in a competitive healthcare system: negotiation 101 for clinicians

**DOI:** 10.1108/JHOM-12-2017-0333

**Published:** 2018-04-09

**Authors:** Robyn Clay-Williams, Andrew Johnson, Paul Lane, Zhicheng Li, Lauren Camilleri, Teresa Winata, Michael Klug

**Affiliations:** 1 Australian Institute of Health Innovation, Macquarie University, Sydney, Australia; 2 Townsville Executive Team, Townsville Hospital and Health Service, Douglas, Australia; 3 Health and Wellbeing Service Group, Townsville Hospital and Health Service, Douglas, Australia; 4 Townsville Skills Centre, Townsville Hospital and Health Service, Douglas, Australia; 5 Clayton Utz, Brisbane, Australia

**Keywords:** Training, Negotiation, Non-technical skills, Resilient health care

## Abstract

**Purpose:**

The purpose of this paper is to evaluate the effectiveness of negotiation training delivered to senior clinicians, managers and executives, by exploring whether staff members implemented negotiation skills in their workplace following the training, and if so, how and when.

**Design/methodology/approach:**

This is a qualitative study involving face-to-face interviews with 18 senior clinicians, managers and executives who completed a two-day intensive negotiation skills training course. Interviews were transcribed verbatim, and inductive interpretive analysis techniques were used to identify common themes. Research setting was a large tertiary care hospital and health service in regional Australia.

**Findings:**

Participants generally reported positive affective and utility reactions to the training, and attempted to implement at least some of the skills in the workplace. The main enabler was provision of a Negotiation Toolkit to assist in preparing and conducting negotiations. The main barrier was lack of time to reflect on the principles and prepare for upcoming negotiations. Participants reported that ongoing skill development and retention were not adequately addressed; suggestions for improving sustainability included provision of refresher training and mentoring.

**Research limitations/implications:**

Limitations include self-reported data, and interview questions positively elicited examples of training translation.

**Practical implications:**

The training was well matched to participant needs, with negotiation a common and daily activity for most healthcare professionals. Implementation of the skills showed potential for improving collaboration and problem solving in the workplace. Practical examples of how the skills were used in the workplace are provided.

**Originality/value:**

To the authors’ knowledge, this is the first international study aimed at evaluating the effectiveness of an integrative bargaining negotiation training program targeting executives, senior clinicians and management staff in a large healthcare organization.

## Introduction

Lack of resources is a perpetual problem in healthcare, an issue unlikely to change with the growing demands of an aging population and the rise in chronic health conditions. Clinicians, especially those with management responsibilities, can often find themselves in situations where in order to maximize resources, they need to negotiate with their colleagues and patients, and sometimes a range of stakeholders including hospital boards, medical committees, politicians, lobbyists, community leaders and business executives (Anastakis, 2003). Corporate firms have long recognized the value of skillful negotiators and invested in training programs to increase the negotiation skills of their managers (ElShenawy, 2010). Intensive negotiation skill training endeavors to improve engagement and collaboration between healthcare professionals, and is an important asset among health care providers (Mellman and Dauer, 2007), yet clinicians receive little or no training in negotiation as part of their medical training.

Much of the healthcare literature on negotiation focuses on conflict resolution, particularly interpersonal conflict between clinicians (Brinkert, 2010; Tabak and Orit, 2007; Almost *et al.*, 2016). In response, standards have been created to support hospital leaders in managing conflict within hospitals so that it does not negatively impact on patient care (The Joint Commission, 2009). While conflict resolution is important, it is only one of the many problems to which negotiation skills can be successfully applied (Lewicki and Hiam, 2007). In normal everyday work, clinicians must negotiate with each other and with managers, to clarify roles and responsibilities and to distribute resources among patient care teams. If this is accomplished effectively, conflict resolution is unlikely to be required.

In the recent Institute for Healthcare Improvement (IHI) white paper, a Framework for Safe, Reliable and Effective Care (Frankel *et al.*, 2017), negotiation was identified as one of the five components of healthcare culture (alongside leadership, accountability, psychological safety and teamwork and communication). While previous research has found benefits in the use of relational narrative methods for negotiation in healthcare (Gerardi, 2015; Scott and Gerardi, 2011), the white paper proposal to implement integrative bargaining approaches is new. Integrative bargaining negotiation offers a framework for increasing value in the organization without extra cost, by promoting integrative “win-win” outcomes.

Research suggests that the effectiveness of negotiation training depends on the design and intensiveness of the program, and that post-training evaluation is crucial (ElShenawy, 2010; Coleman and Lim, 2001). We believe that this is the first international evaluation of integrative bargaining negotiation training in healthcare.

### Description of the intervention

We report the results of an evaluation of intensive negotiation skills training for executive, senior clinician and management staff members at a large tertiary care hospital and health service in regional Australia. Training commenced in February 2015, with 80 staff members completing the program as of May 2016. The two-day course focused on integrative bargaining, training participants to identify “win-win” solutions that can improve efficiency without incurring additional cost. A co-author (MK), who is an internationally recognized consultant and skilled negotiation specialist, facilitated the training. Experiential methods were used, including case studies, groupwork and role-play. Learners were introduced to a Negotiation Toolkit, which contained the seven Elements of Negotiation Scoresheet (where relationship, communication, interests, options, legitimacy, commitments and alternatives are listed and scored), and the Negotiation Worksheet (where theirs/ours interests, Best Alternative to a Negotiated Agreement, and No Deal Option are determined and noted). They were also provided with a set of heuristics – the golden rules – to guide their negotiation practice; examples include: “if you get the process right then the result will look after itself,” “the engine room of negotiation are the interests of the parties,” “high emotion gets in the way of rational negotiation” and “the goal is to establish slow forward controlled momentum.”

The negotiation training aimed to introduce participants to a flexible style of bargaining, where the specific strategy is chosen depending on the relative importance of two aspects: maintaining a positive relationship with the other party, and the outcome of the negotiation itself. Participants were provided with instruction and tools to assist them in assessing their own position, assessing the position of the other party, and selecting a strategy. Practice sessions were conducted in small groups, where participants role-played a series of negotiations based on scenarios provided by the facilitator. Feedback and coaching were provided between scenarios, and each group shared their learning experiences with the class.

**Figure 1:**
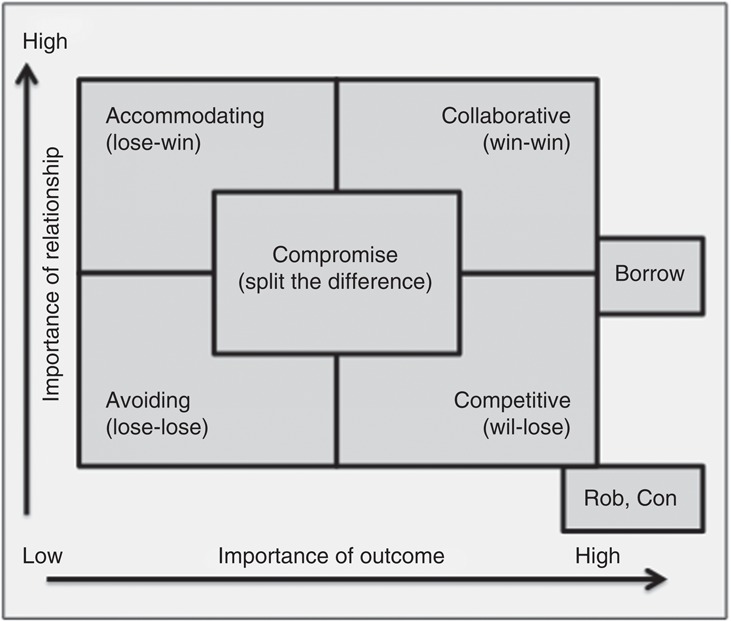
Negotiating style preference grid

Underpinning the ability to choose the best negotiation style for the situation is the need to be aware of the preferred styles of self and others. During the training, participants completed a modified Thomas-Kilmann Conflict Mode Instrument (TKI) self-assessment questionnaire (Kilmann and Thomas, 1977; Shell, 2001), which identified their behavioral preferences for interacting with others. Outcome of the TKI normally places the respondent on a grid with relationship/outcomes axes, and identifies their natural preference for one of five negotiating styles: competitive, collaborative, accommodating, avoiding or compromise (Figure 1). The training used a modified version of the TKI (Lewicki and Hiam, 2006) which identified three additional styles lying “outside” the grid: borrower, con and rob. Further explanation of the styles is shown in Table I.

## Method

### Research objectives

The research aimed to evaluate training effectiveness by investigating whether participants implemented the integrative bargaining skills in the time period since completing the training, and if so, how and when. The study used a qualitative approach, involving face-to-face interviews with participants.

### Selection of participants

All current health service executive, senior clinicians and management personnel who completed the negotiation skills training course (with the exception of the study investigators) were eligible to participate, resulting in a source population of 70 staff members. The sample was stratified prior to random selection of interview participants to ensure that a representative spread was sought from the executive, clinical and management groups.

### Procedure

Each participant was interviewed face-to-face in a private room. Interviews were audio recorded, and professionally transcribed verbatim to prepare them for analysis. Participants were asked about current workplace attitudes, behaviors and processes that may have been impacted by the negotiation training, and their experiences of applying negotiation skills in their workplace. Interviews were conducted at least eight weeks after completion of the training to allow time for the skills to be translated into practice.

### Analysis

Interview data were analyzed in an integrated fashion. Inductive interpretive analysis of transcribed interviews was undertaken to identify key themes relating to the negotiation skills training. Coding the data allowed it to be organized and used to explore connections between data elements and to develop sets of concepts. Once coded, segments of data were linked in a formal fashion to allow themes to emerge and to determine relationships that may exist between different data sets. This is a way of studying real world complex systems such as healthcare.

### Ethical considerations

Ethics board approval for the study was obtained from the XXX Hospital and Health Service Human Research Ethics Committee (HREC/15/XXXX/219) and endorsed by the Macquarie University Human Research Ethics Committee (MQ 5201600280). The study was funded by a small grant from the XXX Hospital and Health Service Research Trust Fund; the funding body had no role in the conduct or reporting of the study.

## Results

### Participants

In total 21 healthcare executives, 20 senior clinicians and 29 managers completed the training. An additional eight learners were no longer employed by the hospital during the study (no information was available on their position), plus two of the learners were part of the research team so not eligible to participate. In all, 18 staff members (6 from each category) who had completed the training, comprising 25 percent of eligible participants, were randomly selected and all agreed to be interviewed. Despite prior stratification, it became evident during the interviews that there was no distinct definition separating managers and executives, and most managers/executives also identified themselves as clinicians; therefore, the results are reported as arising from a single sample (Table II).

**Figure 2:**
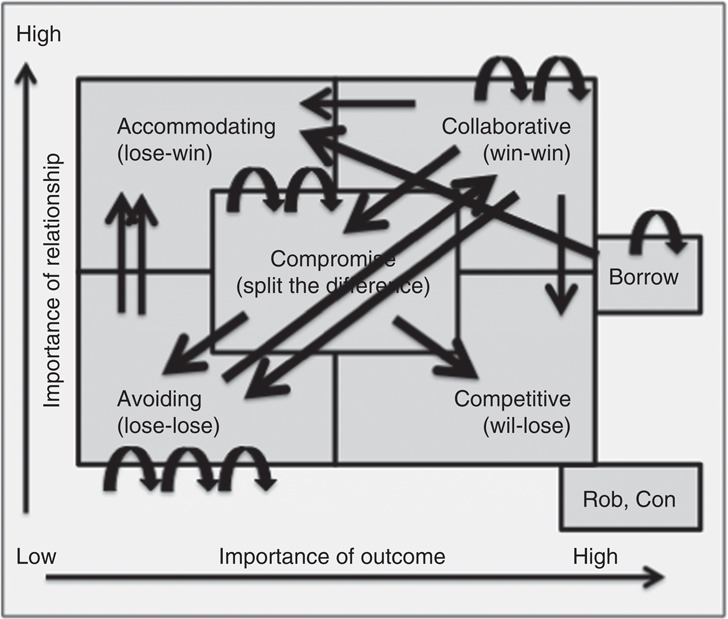
Participant negotiating style movement under pressure

While 8 of the 18 participants completed the course within the previous three months, the remaining 10 participants completed the training between 9 and 15 months previously. In total, 6 participants had either previously completed negotiation training, or had been exposed to negotiating principles; for the remaining 11 participants, the training material was new. Participants were identified by the self-assessment questionnaire to have a natural preference for the following negotiating styles: competitive (*n*=1), collaborative (*n*=6), avoiding (*n*=6), compromise (*n*=3) and borrow (*n*=2). When asked which style they moved to when under pressure, participants were each able to identify a style on the grid, resulting in the following range: competitive (*n*=2), collaborative (*n*=3), avoiding (*n*=4), compromise (*n*=3), accommodating (*n*=5) and borrower (*n*=1). While eight participants stated that they maintained the same style under pressure, the remainder moved on the grid (Figure 2).

### Themes

In all, 6 primary themes and 28 sub-themes emerged from the analysis (see Table III). Primary themes were affective reactions, utility reactions, barriers to implementation, enablers for translation, work practices and sustainability. Sub-themes were: word of mouth, participant view of the facilitator (positive and negative), personal negotiating style preferences, spending time with colleagues, balancing course time and content, participant view of the organization (positive), practical negotiating skills, tools and templates, matching training to user needs (positive and negative), time, negotiating with other parties (within and external to health service), job mobility (negative), provision of the Negotiation Toolkit, standardization of negotiations, integral part of daily work, noticing others, gaining confidence, improved understanding of behavior – self and others, preparation, reduced stress, examples of work practices, few support mechanisms for translating learning, refresher training, coaching and mentors, formal discussion groups, advanced/tailored training, and implementing training more widely.

### Affective and utility reactions

Almost all participants had positive affective and utility reactions to one or more aspects of the training. Participants liked learning about their own negotiating style (and were interested in the styles of others): “I enjoyed that it was group training […] and learning more about my own style, but also recognizing other people’s styles” (Interview No. 2). Most participants expected to gain some practical skills as a result of training: “[…] expect[ed] that I’d come away with some skill or improved skill that would help me in the workplace” (Interview No. 2). For most, the useful components were the practical examples, the structured approach to negotiation and the tools and templates: “[…] just having that framework in your mind around how to do it and how to prepare for it certainly keeps a negotiation on track […]” (Interview No. 5). The key points (or “golden rules”) that work across a broad range of situations, the opportunity for short practice in the groupwork and the insight into personal negotiating style preferences were also considered useful for work. Many participants actively planned to translate the training to the workplace, including arriving at the course with specific examples in mind of where they would use the training when they returned to work: “I actually have quite a few meetings that are quite difficult in lots of ways, where there is a lot of negotiation […] I wanted to be there to learn how to manage those situations better” (Interview No. 9).

### Barriers and enablers

Time was the biggest barrier to employing the skills in the workplace, particularly for executives and clinicians. Most of the participants identified the need to prepare for each negotiation, and felt that finding sufficient time to do so was a challenge: “one of the biggest barriers for me as a clinician is having time to enact what I know” (Interview No. 6). Manager participants who were involved in negotiations with the state health department felt that the unwillingness of the department to negotiate, or the practice of sending negotiators who do not have authority to decide, was also a barrier to both successful negotiation and implementation of the skills: “there are some things that you are able to negotiate but there’s really some things in the structures that we have within [the health department] that you just can’t” (Interview No. 1). These senior managers were both disappointed, and frustrated, that their skills were not able to be fully utilized in these high-level discussions, which were critical for obtaining much-needed hospital and health service resources: “on our really big ticket items there was just no real negotiation to it” (Interview No. 1). They reported that true negotiation seemed to be new to the state department, and that they have yet to develop processes for dealing with health services wishing to negotiate resourcing: “last year we were one of very few [health] service[s] […] to argue or continually argue for stuff […]” (Interview No. 1).

In contrast, within the health service it was sometimes difficult to negotiate with colleagues, as others valued the relationship too much: “[…] when you have a large number of people who are all trying to make sure other people feel OK about a negotiation, actually it becomes real, really difficult [to come to an agreement] […] think within the hospital […] when you’re dealing with people and you invest in a longer term relationship, you tend to be a lot more wanting to please them” (Interview No. 6). Even so, hierarchical relationships and formal reporting structures within the hospital could make it difficult for clinicians to implement the negotiation strategies taught in the training: “[…] for someone in my position, I don’t have the ability to knock on the door or make an appointment with someone two or three levels above me in structure […] the channels of communication within this organization are not open […] what was being talked about in the course is assuming that you’ve got a [more flat gradient]” (Interview No. 13).

The biggest enabler for all participants was provision of the Negotiation Toolkit – a series of worksheets to assist in preparing for negotiations. Some clinicians, managers and executives had the seven Elements of Negotiation Scoresheet, the Negotiation Worksheet and/or the “golden rules” laminated on their desks or pinned on the wall. A further enabler for all participants, associated with the tools, was the potential of standardizing the way negotiations were approached, thereby bringing a common language or understanding to discussions: “I felt that I had a framework that I could use at work when it came to a negotiation and that people that were there from my workplace also could use that framework in a negotiation when we were all in a negotiation with each other, so they know where I’m coming from” (Interview No. 12).

### Work practice

Participants indicated that negotiation formed an integral part of their daily work: “[…] you always use negotiation every day […]” (Interview No. 4). Most participants had employed at least one aspect of the training in their work, and gave one or more specific examples to illustrate where they were approaching their interactions with others differently than in the past, or were more engaged in looking for “win-win” outcomes. Participants in all categories could also point to examples where negotiation was starting to make a positive difference to relationships, personal performance or organizational outcomes. Some participants felt more confident following the training, both personally, and also in their ability to succeed in resolving longstanding or complex problems.

Participants began to notice when others were employing the techniques: “[…] from very, very early on I could see […] the way he was playing the game” (Interview No. 3). For some, it was enjoyable and energizing to negotiate, and to notice how others negotiated: “so in that instance [when negotiating against a difficult opponent] I find negotiation becomes more sporting to me” (Interview No. 3). The examples provided by participants predominantly involved moving from a “win-lose” to a “win-win” situation: “People are trying to work through [their problems] a bit more clearly rather than putting a line in the sand and putting another line in the sand and then digging a trench” (Interview No. 5).

While participants reported seeing the skills utilized at work, this has not yet translated to substantially increased skill or increased intensity of negotiations overall: “Overall shift? Probably not, but certainly in individuals” (Interview No. 5). Nevertheless, while each participant gave examples of where they had personally utilized the skills, a number of participants reported examples where others did not reflect on, or respond to, the training.

Some participants felt more confident following the training, both personally, and also in their ability to succeed in resolving longstanding or complex problems: “I think that I’m probably more tolerant with both conflict and negotiation now. Rather than just looking for the quick result, no matter whether I win it or lose all, I’m happy to take the time and to find a satisfactory result for both parties” (Interview No. 7). One participant explained how negotiations within the health service could be very context driven. For example, to negotiate implementation of the same process across different service groups may require a different approach, depending on the needs of both parties and the degree of cohesion or rapport between them. The skills taught in the training showed how to select the best approach to ensure that during negotiation, the individual needs/wishes of the service group is respected, resulting in preservation of relationships and a win-win outcome. Participants gave examples where they were working differently as a result of the training, including increasing persistence in trying multiple approaches when negotiating around funding: “I learnt to think of it not just one way, put yourself in many shoes […] and if they’ve said no here, how do you come around again […]” (Interview No. 1). For some participants, employing some of the negotiation techniques taught resulted in less stress at work. Having a walk-away position, for example, can lessen the frustration associated with negotiations that do not approach the zone of potential agreement: “a release valve in terms of how I feel about a negotiation” (Interview No. 6). Most participants found it more satisfying in the workplace to have win-win outcomes: “[…] building bridges […] that’s the kind of stuff you do on a daily basis” (Interview No. 17).

In some cases, despite participants not feeling that the course was well matched to their needs, their comments in the interview showed that they have, in fact, gained an improved understanding about the behavior of others in the workplace, and how to better engage with colleagues: “I’m definitely more aware of how other people navigate through the hospital and how the people interact. I’ve definitely become more aware of different relationships and how certain things might play out and reading a room as well” (Interview No. 2). The training has also provided participants with the skills to work as a team: “So the three of us […] two of us are more withdraw types, but we have a strong collaborator […] I’m a little bit more assertive […] [in the meeting] we were, as a group, using all of our skills. We were able to negotiate [our objective] with all the resources that we needed” (Interview No. 2).

Participants believed that preparation was the most important element of skilled negotiation, and many gave specific examples to support this assertion. However, the healthcare workplace infrequently allowed sufficient time for preparation, particularly for clinicians, and this hampered the ability to use the skills for many participants: “The actual time to think and work on the business instead of in the business is very difficult to ring fence and protect” (Interview No. 9).

### Examples of two successful negotiations following the training – Box

Two sisters, one orange: Mary Parker Follett (1940) founded interest based bargaining in the 1920s, with the story of the two sisters arguing over the last orange in the pantry. The sisters reach a traditional distributive compromise whereby they each take half of the orange. Both achieved only half of their desired outcome for their intended purpose: one to extract the juice and the other to use the peel to flavor a cake. The sisters came the realization that both could have had 100 percent of their desired outcome if they had understood each others’ interests, and negotiated on that basis.

In the first example, the hospital identified a critical need for expansion of two facilities: the simulation and clinical skills center, and the formal teaching space. The funding available was capped, and the space available for both projects was constrained by existing physical hospital infrastructure. The combined floor space required by the two projects exceeded available floor space by 50 percent, and available budget by 30 percent. In exploring the interests of each party, the project sponsor was able to identify a shared requirement for small teaching rooms. However, neither party required fulltime access to the facility. Through a flexible planning approach, multipurpose room designs were developed that accommodated the needs of simulation and formal teaching. In addition, a flexible booking arrangement allowed for more effective use of the physical space and gave each party access to more than they had originally required, thereby creating value for all parties.

The second example involves a complex negotiation regarding implementation of the new hospital Integrated Electronic Medical Record (IEMR). The vendor sought to implement a standard IEMR, but the hospital was concerned that this would not meet the hospital needs. The negotiation proceeded over a number of weeks, and involved clinicians, executives, members of the IEMR team, the central health department program director and the vendor. The hospital’s aim for the negotiation was to implement a safe and effective EIMR in the Emergency Department. The vendor’s interest was to implement the IEMR without increasing costs or effort, but there was a significant unstated interest of reputational risk. A single text negotiation document was created by the hospital team, which then drove negotiation with the vendor. A Best Alternative To No Agreement was established by the hospital as the intent to walk away from the deal. The vendor opened the negotiation with the position that they would not be able to vary the installation due to costs, etc., but the hospital team was able to leverage off reputational interest to achieve their aims. The hospital team established five key issues to be addressed, recognizing that they would need to compromise on these to achieve a negotiated outcome, and they were prepared to offer suitable alternative options to the vendor. Two weeks from the “go live” for the IEMR, the vendor agreed to four of the five demands, and the fifth was negotiated to follow in the coming months. Due to cohesion developed through the negotiation process, the hospital team was able to implement the solution within a shortened timeframe.

### Sustainability

There was some overlap between ideas for improving sustainability and enablers for translating the training into the workplace, in that having processes for sustainability in place was considered to aid training transfer. Despite high levels of engagement, participant recall of specific learning points was variable, and was more dependent on whether the participant was using the skills in practice than how long since they had trained. However, all participants could recall their preference for negotiating style, and the style they reverted to under pressure, when shown a picture of the grid in Figure 1.

Most participants were disappointed to find that, following the training, there were few support mechanisms to assist in implementing the skills in the workplace: “you come back to the organization […] excited […] [but] there was nothing else, no follow up” (Interview No. 1). Reciprocity from colleagues, particularly if they had also completed the training, was important for assisting participants in applying the skills: “So I think […] [for example] that potentially setting an agreement with the group and then going okay so in our monthly meetings let’s bring in case studies and work with it as a group to do that transition of learning” (Interview No. 15). Many participants raised the need for assistance in translating negotiation skills to the workplace, and in sustaining learning. Because most came from senior or managerial positions, participants were also cognizant of the difficulty of sustaining learning: “Because we’re very busy. There is nobody that works in health that isn’t very busy, whether they’re clinicians or managers or whatever. The problem is you go into a course, which was exceptional. It was a very good course. But it’s how do you keep that fresh in people’s minds without that being onerous for them, because we have competing priorities all the time” (Interview No. 16).

Most participants felt that refresher training would be useful, and had strong, but quite varied (and sometimes conflicting) views on how this should be delivered: options included repeating the course every couple of years, completing an abbreviated version of the training (half to one day), and completing monthly sessions with other interested colleagues to work through specific practical problems: “I think you’d probably get a summative effect from going back and refreshing” (Interview No. 6). Participants also felt that coaching, whether in the form of a mentor, or a person to call to discuss difficult or complex situations, would be helpful and enhance learning and training transfer: “perhaps having a mentor, I think, would be beneficial, a senior mentor that you can go to a month after training, and then maybe six months, and then maybe 12 months, so that you can discuss – keep it fresh in your mind and discuss your progress and potential issues” (Interview No. 7).

Some participants felt that having a mentor, and then becoming as mentor as experience was gained, might mean that refresher training was not required: “You might not need [refresher training] if you had that mentor to build it in and then if you became a mentor for somebody else” (Interview No. 4). Others felt that just having someone to call if needed was a better option than a formal mentor: “I’ve never been good with the mentor concept, to be honest” (Interview No. 5). Certainly, some colleagues looked to others for support as they learned the new techniques: “Having people around that knew the resource well [would make it easy to apply the skills in the workplace] […] after I did [the training] I spent some time […] had more contact time with [an executive who had completed the training earlier, who provided a role model]” (Interview No. 9). Other refresher training options included a subset of refresher training at 18-24 months, where participants could choose to refresh on technical or process issues, practical implementation problems, or both.

The need for formal discussion groups was also raised by a number of participants: “maybe a small work group of peers […] getting together and just having a half hour or so session […]” (Interview No. 5). Practice was considered to be critical for sustainability: “the more times you practise using the template, and the more times you practise a different negotiation style, the more likely that is to start to impact on your day-to-day [work]” (Interview No. 6). Participants felt that if there was a more regular discussion, the principles were more likely to be assimilated: “I think if we […] talk about it regularly, it would stick better in my mind […] if we did use it all the time, it’d be a lot easier to put into the workplace” (Interview No. 4). In general, however, participants who regularly used the tools reported that by 12 months in: “it’s becoming a more unconscious skill” (Interview No. 9).

Some participants also suggested the need for advanced training, for those already versed in the basic skills but needing to work through more complex, sometimes healthcare-specific, scenarios. Some participants also felt that the training needed to be applied more widely across the health service: “I think the knowledge and education does need to be shared broadly” (Interview No. 9).

## Discussion

In healthcare, authority gradients are flat (and sometimes reversed), and clinicians and managers frequently need to convince others over which they have no authority of the need to pursue a particular course of action. Successful outcomes often depend on establishing a series of small or large ongoing agreements between individuals or craft groups, and negotiation skills are crucial. IHI suggest that “Health care teams should commit to using collaborative negotiation whenever possible. This is the only negotiation approach that yields workable solutions that manage resources, provide the best options for patients, and preserve the relationships between parties” (Frankel *et al.*, 2017). While we agree that this is desirable, it is important to note in that, in our study, participants reported success using a variety of negotiation strategies. Despite the large variety of negotiating styles that were found among the executives, managers and clinicians in our study, the importance of negotiation in the workplace, and the need for tools and techniques to assist in negotiating successfully in everyday work, were universally accepted, and we found considerable evidence that the skills are being applied to solve both problems ranging from small to large and complex (see Box for two examples). These findings confirm what we know from the literature, i.e. that content relevance and opportunity to perform have a strong to moderate relationship with transfer of training to the workplace (Burke and Hutchins, 2007), and that utility reactions correlate with learning and on the job performance (Alliger *et al.*, 1977; Ruona *et al.*, 2002).

An unanticipated benefit of the training was an opportunity for participants to spend two full days interacting with colleagues. Some participants found this valuable for networking; others found it useful to gain a better understanding of how other parts of the hospital function. Research has found that creating cohesive collaborative networks is associated with better patient care (Cunningham *et al.*, 2012), yet it is only during training that many professionals have a chance to get to know on another.

A number of participants drew heavily on the behavior grid, both to provide self-insight and also to enable insight into the behavior of others. These insights were useful not just in planning formal negotiations, but also in the informal negotiations that form everyday communication in healthcare, suggesting that there might be a role for this type of learning in current teamwork communication training. In their normal work, most participants clustered around the collaborative/compromise/avoid behaviors. Shell (2001) found that clinical professionals in the health care field systematically report both less competitive and more accommodating TKI scores than do executives in more traditional businesses. In contrast, our study found that behavior most perceived as typical of healthcare professionals – accommodating – was only favored when participants were under stress (and, even then, by less than one-third of participants). Tabak and Orit (2007) also found that healthcare professionals’ behaviors moved from collaborating and competitive toward accommodating and avoiding when under stress, however, our study showed the reality to be more complex with no consistent pattern of behavior change reported by participants to be associated with stress.

Those enrolled in the training who were new to their job did not seem to gain as much benefit as other participants, possibly because utility of the material was not yet established for these participants. Self-selection into training may be a viable option with this group of professionals, as participants were generally very aware of their personal limitations and the areas where they needed to learn or practice. Given the time pressures of clinicians, there might be value in modular training, where, for example, theory delivered via didactic modules or pre-reading is followed by face-to-face training sessions interspersed with practice implementing the ideas in the workplace. Clinicians might also benefit more than executives from tailored training, especially the inclusion of more clinically relevant scenarios and group practice – perhaps in a similar format to current simulation team training (Baker *et al.*, 2005). Practicing clinicians also seemed generally to be more interested in the behavioral aspects of the training when compared with executives (who seemed more interested in the negotiation processes), perhaps because they interact with greater variety of individuals in their day-to-day care of patients. Clinicians also generally had a greater focus on learning through teaching others, again perhaps because this aligns more closely with how they teach and learn technical skills and non-technical workplace behaviors.

Instituting formal refresher sessions or other ongoing training strategy is important for sustaining positive changes to the way people work. Participants kept their course notes, and many said that they intended to reflect on the material, but admitted they had not got around to it. Lack of time to reflect on the training, to prepare for negotiations, and to apply the negotiation techniques, was a large and frequently reported barrier to implementing the skills in the workplace. There is no protected “thinking time” in the participants’ day, and, even where scheduled, it can be overridden by the more overt and seemingly urgent needs of others. Even at the highest level, time use of executives seems frequently to be controlled by others.

Noticing others using the skills in the workplace provided both a revision of the principles, and also, when those observed were successful, positive reinforcement of the value of using the skills. Laminating the material for mounting on wall or desk also enabled reflection or revision on the principles, and a visual “aide memoire” to use them. The Negotiation Toolkit, in particular, proved to be very useful, with more than half of the participants saying that they used (or referred to) at least one element of the kit when preparing for negotiations. Previous research has found that on average only from 10 percent (Fitzpatrick, 2001) to 47 percent (Saks and Belcourt, 2006) of what is learned in training is applied at work. Unlike many training courses, the majority of participants in the negotiation skills training appeared to have assimilated at least some of the principles into their everyday work. Even those who trained most recently talked about their attitudes to solving disagreements, involving people or resources, in ways that showed the training was taking effect. Even those who did not like the training, or think it particularly useful, reported adopting some of the principles.

Annual negotiations between the health service and the state health department, particularly where they involve funding, can be protracted. While the health department pays lip service to the concept of negotiation, the participants were able to easily recognize that the stated intent to collaborate and negotiate was little more than an aspirational goal. Transfer climate has been found to have a major influence on whether training is transferred (Goldstein and Ford, 2002; Rouiller and Goldstein, 1993; Tracey *et al.*, 1995; Warr *et al.*, 1999), and participants become frustrated when provided with a skill then prevented from using it. Implementing the training in the health department, therefore, would improve utility of the training at the health service level.

### Implications for the organization

Our findings suggest that negotiations should be face-to-face, even where this involves travel costs. There seems to be a trade-off between short and long-term gains – long-term gains preserve relationships, but the organization should be willing to accept that there may be no obvious immediate outcome. Standardizing the negotiation processes across the health service (and, ideally, the state health department) and implementing training more widely is likely to assist participants in trying to adopt the skills in the workplace, including those who move between jobs or departments.

Time was identified as a major barrier to successful development of negotiation skills. Consideration should be given on whether it is possible to simplify the training process or break it into shorter modules, and time should be allocated for staff to implement the processes at work (particularly in the first few months following training).

Our findings identified a need for an organization wide strategy underpinning the training, including formal support for translating the skills to the workplace and for ongoing sustainability of learning. The training and skills development should be formally aligned to other workplace key performance indicators.

### Implications the broader knowledge base

We know, for the first time, that integrative negotiation methods can be taught to healthcare professionals, and that integrative bargaining skills are reported by the majority of healthcare workers to be a valuable skill for the workplace. Our findings suggest that almost all healthcare executives, senior clinicians and managers are able to apply practical bargaining skills to solve workplace problems after only two days of basic negotiation training.

### Limitations

Limitations of this study are that the data are self-reported, with all the attendant biases. In addition, participant views of the facilitator were polarized, with the majority displaying an intensely positive affective reaction, and this “halo” effect (Nisbett and Wilson, 1977) may have colored their assessment of the training and its efficacy. In addition, from analysis of participant responses, the TKI-based questionnaire that was delivered in the training appears to be quite context sensitive. Despite this, questions asked about reactions in non-healthcare context are used to derive a behavior assessment that the participant is then encouraged to apply in their healthcare workplace. While the TKI is a validated instrument (Thomas, 1974), and the work of Lewicki *et al.* (Lewicki and Hiam, 2006, 2007) provides a link from the TKI to the behavior grid used on course, the questionnaire used in the training appeared to be based on different questions to the TKI. It was not possible from the literature, therefore, to determine validity of the course questionnaire for use in determining negotiating style preference. For these reasons, outcomes of the questionnaire should be treated with caution. While the instrument was likely offered by the facilitator merely as a tool to encourage self-insight, the assessments were accepted by many participants as fact, and used to drive judgments about self and others at work. Perhaps because many participants treated the results of the questionnaire as fact, the outcome for each participant in some cases appeared to bias their reactions to the training.

The nature of the interview encouraged participants to report where they had used the training, and is therefore likely to have had a positive bias on the number of reported examples. A number of interviewees described negotiations involving human resources situations; while these negotiations broadly involved the same processes and similar stories of win-win successes as the negotiations around other topics, they have not been included in this paper to protect identity of participants.

### Conclusion

Our study found that participants generally valued negotiation training and were applying the principles in the workplace. Negotiation is common in clinical everyday work, and is not limited to situations requiring conflict resolution. Uptake and translation of integrative negotiation skills to the workplace appears to be high in comparison with other health service training. Interviewing more than a quarter of participants, including participants who had completed training both recently and between 9 and 15 months previously, and randomly selecting those for interview from a stratified sample, all contributed to high confidence that our findings are valid.

## Figures and Tables

**Table I:** Negotiating styles

Competitive	The competitive style reflects a high importance for outcome, but a low concern for the relationship. A person adopting this position will take, engage, and use the rules to their advantage. It can be characterized as a win-lose strategy
Collaborative	The collaborative style reflects a high importance for outcome, and a high concern for the relationship. A person taking this position will give more than they take, engage rather than withdraw, and have limited tolerance for rules. This strategy will usually attempt to create additional value as part of the negotiation. To achieve this win-win outcome, both parties must find ways to get what they each need while enhancing the relationship
Accommodating	The accommodating style reflects a low importance for outcome, but a high concern for the relationship. A person taking this position will give, they will engage and they will accept the rules. It can be characterized as a lose-to-win strategy, where the accommodating party will back-off or give-in to preserve the relationship
Avoiding	The avoiding style reflects a low regard for both outcome and relationship. A person adopting this position tends to give more than they take, and withdraw. They may think outside the square and often have an exceptional eye for detail. While this can be characterized as a lose-lose strategy, which usually involves ignoring the problem or walking away from any interaction with the other party, the only difference between an avoiding and collaborative position is the degree of engagement
Compromise	The compromise style is a combination of elements of the other styles. Relationship and outcomes are both considered important, but rather than creating additional value so that both parties can get what they need, this style will “split the difference”. It is a useful fall back position where the outcome is important, but there is insufficient time or resources to pursue the collaborative option, and avoids the competitive “win at all costs” that will destroy the relationship
Borrower	The borrower style has 3 components: outgoing, giving and with a high focus on detail, practices and procedures. A person taking this position tends not to observe the rule of reciprocity and can be quite closed with information sharing. Whilst not unethical, the borrower can be viewed (often wrongly) with suspicion by co-workers as sneaky or untrustworthy
Con	The con is an unethical style that reflects a high importance for outcome, and deliberately inflicts damage on the relationship in order to achieve that outcome. This style should never be used, but it is important to recognize if the other party is willing to behave unethically so that the negotiation can be halted
Rob	To rob is an unethical style that reflects a high importance for outcome, and displays greater intent to damage the relationship than the con style. It is important to recognize if the other party is willing to behave unethically so that the negotiation can be halted

**Table II:** Demographic characteristics of study participants

Characteristics	Participants (*n*=18)
*Gender*	
Male	8
Female	10
*Age (years)*	
20-30	1
31-40	6
41-50	10
51-60	1
*Time in profession (years)*	
<1	5
1-2	6
3-4	5
⩾5	2
*Duration at organization (years)*	
<1	1
1-5	7
6-10	5
11-15	3
⩾16	2

**Table III:** Main themes

Affective reactions	Word of mouth
	Participant view of the facilitator (positive and negative)
	Personal negotiating style preferences
	Spending time with colleagues
	Balancing course time and content
	Participant view of the organization (positive)
Utility reactions	Practical negotiating skills
	Tools and templates
	Matching training to user needs (positive and negative)
Barriers	Time for preparation for negotiations
	Negotiating with other parties (external to health service)
	Negotiating with other parties (within health service)
	Job mobility (negative)
Enablers	Provision of the Negotiation Toolkit
	Standardization of negotiations
Work practices	Integral part of daily work
	Noticing others
	Gaining confidence
	Improved understanding of behavior – self and others
	Preparation
	Reduced stress
	Examples of work practices
Sustainability	Few support mechanisms for translating learning
	Refresher training
	Coaching and mentors
	Formal discussion groups
	Advanced/Tailored training
	Implementing training more widely
